# Anti-DFS70 antibodies detected by specific methods in patients with thrombosis or recurrent pregnancy loss: no evidence of an association

**DOI:** 10.1038/s41598-020-64550-y

**Published:** 2020-05-08

**Authors:** Nicola Bizzaro, Giampaola Pesce, Maria Teresa Trevisan, Manuela Marchiano, Luigi Cinquanta, Maria Infantino, Giusy Paura, Marilina Tampoia, Maria Grazia Alessio, Giulia Previtali, Magda Marchese, Clelia Zullo, Danilo Villalta, Ignazio Brusca, Mario Laneve, Caterina Castiglione, Teresa Carbone, Carmela Curcio, Laura Invernizzi, Fabrizio Montecucco, Daniele Saverino, Fabio Ferretti, Brunetta Porcelli

**Affiliations:** 1 Laboratorio di Patologia Clinica, Ospedale San Antonio, Azienda Sanitaria Universitaria Integrata di Udine, Tolmezzo, Italy; 2 Laboratorio Diagnostico di Autoimmunologia, IRCCS Ospedale Policlinico San Martino, Genova, Italy; 3 Dipartimento di Medicina Interna e Specialità Mediche (DIMI), Università degli Studi di Genova, Genova, Italy; 4 Unità Operativa di Laboratorio, Ospedale G. Fracastoro, Verona, Italy; 5 Laboratorio centralizzato SDN Spa, Gruppo SYNLAB, Salerno, Italy; 6 Laboratorio Immunologia Allergologia, Dipartimento di Medicina di Laboratorio, Ospedale San Giovanni di Dio, Azienda Usl Toscana Centro, Firenze, Italy; 7 Patologia Clinica, AOU San Giovanni di Dio e Ruggi d’Aragona, Salerno, Italy; 8 Laboratorio di Autoimmunologia UOC di Patologia Clinica Universitaria, Azienda Ospedaliero-Universitaria, Policlinico di Bari, Italy; 9 Laboratorio Analisi Chimico Cliniche, ASST Papa Giovanni XXIII, Bergamo, Italy; 10 Servizio di Patologia Clinica, Ospedale Santa Maria Delle Grazie, Asl Napoli 2 Nord, Pozzuoli-Napoli, Italy; 11 UOC di Procreazione Medicalmente Assistita, Ospedale S.M. delle Grazie, Pozzuoli, Italy; 12 Immunologia e Allergologia, Presidio Ospedaliero S. Maria degli Angeli, Pordenone, Italy; 13 Patologia Clinica, Ospedale Buccheri La Ferla FBF, Palermo, Italy; 14 Patologia Clinica, Presidio Ospedaliero Annunziata, Taranto, Italy; 15 Laboratorio Patologia Clinica P.O. Santo Spirito, Pescara, Italy; 16 IReL – Istituto Reumatologico Lucano – Ospedale San Carlo, Potenza, Italy; 17 Ostetricia e Ginecologia, Ospedale San Carlo, Potenza, Italy; 18 Chirurgia Vascolare, Ospedale G. Fracastoro, Verona, Italy; 19 Clinica di Medicina Interna 1, Dipartimento di Medicina Interna e Centro di Eccellenza per le Ricerche Biomediche (CEBR), Università degli Studi di Genova, Genoa, Italy; 20 IRCCS Ospedale Policlinico San Martino, Genoa, Italy; 21 Dipartimento di Medicina Sperimentale DiMES, Università degli Studi di Genova, Genoa, Italy; 22 Dipartimento di Medicina, Chirurgia e Neuroscienze, Università degli Studi di Siena, Siena, Italy; 23 Dipartimento Biotecnologie Mediche, Università degli Studi di Siena, UOC Laboratorio Patologia Clinica, Policlinico S. Maria alle Scotte, AOU Senese, Siena, Italy

**Keywords:** Immunology, Rheumatology

## Abstract

A dense fine speckled pattern (DFS) caused by antibodies to the DFS70 kDa nuclear protein is a relatively common finding while testing for anti-nuclear antibodies (ANA) by indirect immunofluorescence (IIF) on HEp-2 cells. However, despite many efforts and numerous studies, the clinical significance of anti-DFS70 antibodies is still unknown as they can be found in patients with various disorders and even in healthy subjects. In this study we aimed at verifying whether these antibodies are associated with thrombotic events or with unexplained recurrent pregnancy loss (RPL). We studied 443 patients with venous or arterial thrombosis or RPL and 244 controls by IIF on HEp-2 cells and by a DFS70-specific chemiluminescent immunoassay (CIA). The DFS pattern was observed in IIF in 31/443 (7.0%) patients and in 6/244 (2.5%) controls (*p* = 0.01) while anti-DFS70 specific antibodies were detected by CIA in 11 (2.5%) patients and in one (0.4%) control (*p* = 0.06). Positive samples, either by IIF or by CIA, were then assayed by a second DFS70-specific line-immunoassay (LIA) method: 83.3% of the CIA positive samples were confirmed DFS70 positive versus only 29.7% of the IIF positive samples. These findings show that IIF overestimates anti-DFS70 antibody frequency and that results obtained by specific CIA and LIA assays do not indicate that venous or arterial thrombosis or RPL are linked to a higher prevalence of anti-DFS70 antibodies.

## Introduction

Testing for antinuclear antibodies (ANA) by indirect immunofluorescence (IIF) on HEp-2 cells is a useful tool for the serological diagnosis of systemic autoimmune rheumatic diseases. Among the numerous ANA-IIF patterns, a distinctive one is the dense fine speckled (AC-2 of the ICAP standardized nomenclature) which is characterized by a nuclear speckled fluorescence of interphase cells and a positive chromatin staining in metaphase cells. The autoantibodies recognize the 70 kDa dense fine speckled protein (DFS70) (also known as the lens epithelium derived growth factor - LEDGFp75), a survival protein implicated in cellular protection against oxidative DNA damage and resistance to stress-induced cell death^1–3^. The protein may be over-expressed or altered during inflammation, thus stimulating autoantibody responses^4,5^. Anti-DFS70 antibodies have aroused in recent years a growing interest due to their frequency, which is around 2–4% of all ANA tests performed in the routine work up^6,7^, and especially because their clinical and biological significance remains undefined.

Initially described in patients with ocular, cutaneous or allergic diseases, soon it became evident that anti-DFS70 antibodies could be found in patients with many other disorders of autoimmune or non-autoimmune origin^6–13^, and even at a higher frequency in healthy subjects^10,14,15^. Thus, despite many efforts and numerous studies, the search for their association with a specific disease has been frustrating so far.

To further complicate matters in the quest to establish their possible clinical association, it has been seen that their prevalence reported in different cohorts of diseased subjects as well as in healthy individuals is dependent on the detection method employed. For instance, their recognition by IIF is not standardized, being highly related to the characteristics (brand) of the HEp-2 substrates used and to the experience of the readers^16,17^. This is confirmed by an international internet-based interpretative survey conducted by Bentow *et al*., who have shown that in samples with isolated anti-DFS70 positivity, the DFS70 pattern was correctly identified by only 50% of the participants^18^. No help comes from automated computer-aided digital systems for reading and interpreting ANA on HEp-2 cells. In one study it was found that these systems are able to recognize 85% of the homogeneous patterns and 78% of the speckled patterns, but none of the samples with the DFS70 pattern that are classified either as homogeneous or speckled^19^.

For these reasons, there is now a widespread consensus that for their proper identification a more specific method such as chemiluminescence^20,21^, line-immunoassay^22^, immunoabsorption^21,23^, or DFS70 knocked-out HEp-2 cells^24,25^ is needed for confirmation of a DFS70-like pattern on the ANA-IIF test.

Interestingly, very recently it has been suggested that anti-DFS70 antibodies may be associated with thrombotic events and that their presence might be indicative of a thrombophilic status. Marlet and coworkers^26^ studied two groups of patients: the first one consisted of 421 consecutive patients presenting a DFS70-like pattern at the routine ANA IIF screening test, referred by internists to diagnose connective tissue diseases or by hematologists to investigate a history of thrombosis. Unexpectedly, they found that 13.1% of their patients had had a thrombotic event or obstetric complications. This finding prompted the authors to study a second cohort of 63 patients with a history of confirmed idiopathic arterial thrombosis (myocardial infarction or ischemic stroke) or venous thromboembolism (deep vein thrombosis or pulmonary embolism), and patients with obstetric complications (≥3 miscarriages before the 10^th^ week of gestation, or fetal death after the 10^th^ week of gestation, or premature birth with eclampsia before the 34^th^ week of gestation); 11.1% of patients in this group displayed a DFS70-like pattern. However, though the study was well designed and conducted by expert researchers, results were questioned^27^ because identification of anti-DFS70 antibodies was based only on the IIF pattern and the specificity of the immunofluorescence finding was not confirmed with a specific method.

Therefore, this study was undertaken to verify on a larger series of patients whether an association exists between thrombotic events (arterial or venous thrombosis) or recurrent pregnancy loss (RPL) and anti-DFS70 antibodies and whether the high prevalence of anti-DFS70 antibodies in these disease groups described by Marlet using the IIF method could be confirmed by DFS70 specific methods. In addition, since heparin plays a role in the trafficking and function of DFS70/LEDGFp75^28^ and no previous studies have investigated the possible relationship between anti-DFS70 antibody expression and heparin, we decided to include in the study a cohort of patients and controls treated with heparin.

## Materials and methods

### Patients

In this multicenter collaborative study of the Study Group on Autoimmune Diseases of the Italian Society of Clinical Pathology and Laboratory Medicine we collected sera from 443 subjects who had suffered a thrombotic event, including 116 with primary anti-phospholipid syndrome (APS) classified according to the international criteria of Sydney, 130 with venous thromboembolism (VTE) (116 with deep vein thrombosis and 14 with pulmonary embolism; 79 on oral anticoagulation treatment - OAT), 69 with arterial thrombosis (7 on OAT), 27 with arterial or venous thrombosis on heparin therapy. Patients were randomly selected among those attending the department of Internal Medicine or Thrombosis Centers. Selection criteria were based on the clinical diagnosis, in the absence of a known cause of thrombosis. Therefore, patients recognizing a specific post-traumatic or post-surgical thrombotic event, patients with cancer and cases in which thrombosis was the result of prolonged immobilization, were excluded to avoid possible confounding factors. We also included a cohort of 101 women with unexplained intrauterine RPL defined according to the European Society of Human Reproduction and Embryology (ESHRE) guidelines^29^ (>2 spontaneous pregnancy losses before the 24^th^ week of gestation excluding ectopic and molar pregnancies). Patients were recruited in the hospital departments of Obstetrics and Gynecology or in Centers for Medically Assisted Procreation. Besides the group with APS, the other patients had no history of autoimmune diseases.

The control group comprised 99 serum samples from patients with atrial fibrillation on OAT; 45 subjects without thrombosis who were undergoing prophylactic heparin treatment before orthopedic surgery; and 100 healthy blood donors without a history of thrombosis or of RPL. Inclusion of a group of patients and controls on heparin treatment was motivated by the overexpression of the DFS70/LEDGFp75 protein induced by heparin. DFS70/LEDGFp75 has a high affinity for heparin and protects it from proteolytic degradation and from heat and acid inactivation^28^.

The median age of the 443 patients was 53 years (range, 21–92); that of the controls was 68 years (range, 21–92). Among patients, 314 (70.8%) were women, with a mean age of 51.2 years (median 49); in healthy controls, women were 71 (71%) with a mean age of 51.6 years and a median age of 51 (Table 1).

**Table 1:** Demographic characteristics of patients and controls.

	*Patients*	*Controls*	*P*
No.	443	244	—
Median age, years (range)	53 (21–92)	68 (21–92)	<0.0001
Gender F/M	314/129	142/102	—
% females (patients *vs*. healthy)	70.8	71.0	0.44
Median age females (patients *vs*. healthy)	49	51	0.39

Data for anticardiolipin antibodies (aCL), anti-β2 glycoprotein I (β2GPI) antibodies and lupus anticoagulant (LAC) were obtained by the referring clinicians or patients records: data on tests for aCL were available in 294 patients (116 with APS, 101 with RPL, 22 with arterial thrombosis, 13 with thrombosis on heparin and 45 with VTE), in 239 patients for anti-β2GPI, and in 173 patients for LAC. The presence of genetic abnormalities or defects predisposing to thrombosis (Factor V Leiden; prothrombin G20210A mutation; MTHFR mutation with hyperhomocysteinemia; antithrombin III, protein S and / or protein C deficiency) was retrieved from clinical records and was available in 167 patients with thrombosis or recurrent pregnancy loss.

### Ethical approval

All procedures performed in studies involving human participants were in accordance with the ethical standards of the institutional and/or national research committee (Ethic Committee of the Hospital of Taranto/Brindisi (Authorization no. 42369) and by the Ethic Committee of Bergamo Hospital (Authorization no. 45104/III-1) for the healthy donors) and with the 1964 Helsinki declaration and its later amendments or comparable ethical standards.

### Informed consent

Informed consent was obtained from all individual participants included in the study. Records and information were anonymized and de-identified prior to analysis, according to the Declaration of Helsinki and to the Italian legislation (Authorization of the Privacy Guarantor No. 9, December 12th, 2013).

### Research involving human participants and/or animals

This article does not contain any studies with animals performed by any of the authors.

## Methods

All samples were tested both by IIF on HEp-2 cells (Euroimmun, Luebeck, Germany) and by a DFS70-specific chemiluminescence assay (CIA). The IIF test was performed in the Laboratory of the University of Genoa on HEp-2 cells at the screening dilution of 1:80 and the interpretation of the ANA-IIF test was done by two observers with more than 20-year experience in pattern reading. Specific research for anti-DFS70 antibodies was performed in the Laboratory of San Bonifacio (Verona) using the QUANTA Flash DFS70 CIA (Inova Diagnostics, San Diego, CA) on the Bioflash instrument (Biokit, Barcelona, Spain). Based on a previous study^21^ the cutoff determined by receiver operating characteristic (ROC) curves was set at 20 chemiluminescent units (CU) which is identical to the cutoff recommended by the manufacturer. Serological analyses were performed blinded to clinical data.

### Statistical analysis

Descriptive statistics were used to analyse the variables’ main characteristics. Once the assumption of normal distribution for the quantitative measures was rejected by the Kolmogorov-Smirnov test, non-parametric tests were chosen to verify the research hypothesis. The one-sided Fisher’s exact test was used to assess the association between the categories of two dichotomous variables and the Mann-Whitney U test was

used to compare the median ages for the two groups of subjects (cases *vs*. controls). Statistical analyses were performed with SPSS-IBM v23 and the level of significance was set at p < 0.05.

## Results

**Figure 1:**
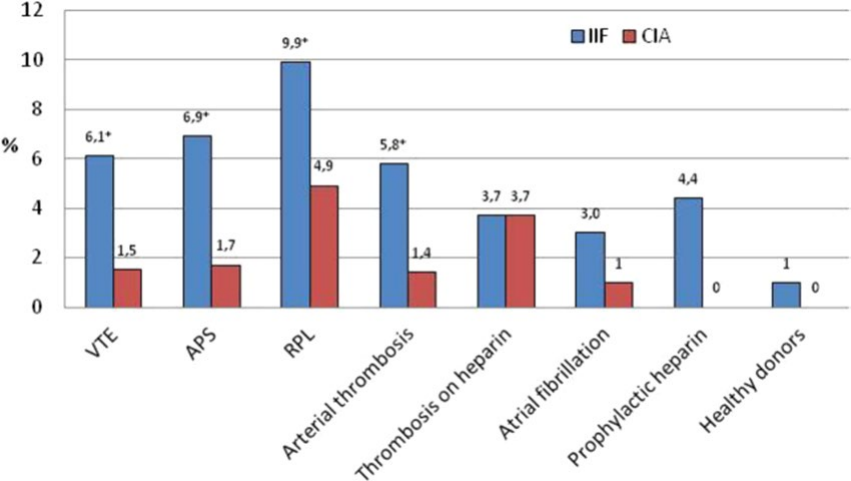
Prevalence (%) of anti-DFS70 antibodies detected by indirect immunofluorescence (IIF) and by a chemiluminescence method (CIA) in patients and controls (VTE, venous thromboembolism; APS, anti-phospholipid syndrome; RPL, recurrent pregnancy loss). Significant differences are marked by *.

The dense fine speckled pattern was observed in IIF in 31/443 (7.0%) patients (27 women and 4 males) at a titer ranging from 1:80 to 1:2560: eight had APS, eight had VTE, four had arterial thrombosis, ten had RPL, one had thrombosis in treatment with heparin, and in 6/244 (2.5%; *p* = 0.01) controls (three with atrial fibrillation, two on prophylactic heparin and in one healthy donor). Conversely, anti-DFS70 antibodies were detected by CIA in 11/443 (2.5%) patients (9 women and 2 males; mean value, 118 CU; range, 23.9-450): two with APS, two with VTE, one with arterial thrombosis, five with RPL, one with thrombosis on treatment with heparin, and in 1/244 (0.4%) control with atrial fibrillation (43.8 CU) (*p* = 0.06) (Fig. 1).

**Figure 2:**
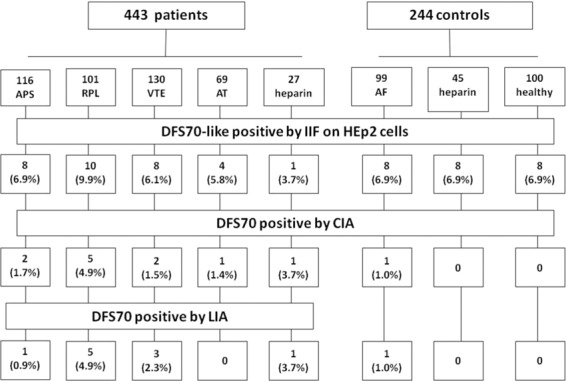
Flow diagram of study results showing number and percentage of samples that were found positive for anti-DFS70 antibodies by the methods used (IIF, indirect immunofluorescence on HEp2 cells; CIA, chemoluminescence immunoassay; LIA, line-immunoassay; APS, anti-phospholipid syndrome; RPL, recurrent pregnancy loss; VTE, venous thromboembolism; AT, arterial thrombosis; AT, atrial fibrillation).

Twenty two patients and five controls were positive only by IIF; two patients were positive only by CIA (at 24 and 33 CU); and nine patients and one control were positive by both methods. Thus, the prevalence rate of positive results by IIF was nearly 3-fold higher than the prevalence detected by CIA. Distribution of results per clinical diagnosis and method are summarized in Table 2. To verify these findings, all positive samples (either by IIF or by CIA) were then tested by a DFS70-specific line-immunoassay (LIA) (ANA Profile 3 plus DFS70 - Euroimmun). All anti-DFS70 IIF + CIA + samples (9 patients and one control) were confirmed positive for anti-DFS70 antibodies by LIA). Instead, of the 27 samples (22 patients and five controls) that were IIF + and CIA−, 25 (92.6%) were negative by LIA as were the two CIA + IIF- samples (Table 3 and Fig. 2).

**Table 2:** Distribution of anti-DFS70 antibodies by the detection method, in patients with thrombosis or recurrent pregnancy loss (RPL) and in controls.

	no.	IIF +	CIA +	*P*	IIF + / CIA−	IIF− / CIA +	IIF + / CIA +	Total +
**Thrombosis/RPL group**								
VTE	130	8 (6.1%)	2 (1.5%)	0.001*	6 (4.6%)	0	2 (1.5%)	8 (6.1%)
Arterial thrombosis	69	4 (5.8%)	1 (1.4%)	0.04*	4 (5.8%)	1 (1.4%)	0	5 (7.2%)
APS	116	8 (6.9%)	2 (1.7%)	0.03*	7 (6.0%)	1 (0.9%)	1 (0.9%)	9 (7.7%)
RPL	101	10 (9.9%)	5 (4.9%)	0.02*	5 (5.0%)	0	5 (5.0%)	10 (9.9%)
Thrombosis on heparin	27	1 (3.7%)	1 (3.7%)	1.0	0	0	1 (3.7%)	1 (3.7%)
**Total**	**443**	**31 (7.0%)**	**11 (2.5%)**	0.0001*	**22 (4.9%)**	**2 (0.5%)**	**9 (2.0%)**	**33 (7.4%)**
**Control group**								
Atrial fibrillation	99	3 (3.0%)	1 (1.0%)	0.15	2 (2.0%)	0	1 (1.0%)	3 (3.0%)
Prophylactic heparin	45	2 (4.4%)	0	0.15	2 (4.4%)	0	0	2 (4.4%)
Healthy donors	100	1 (1.0%)	0	0.31	1 (1.0%)	0	0	1 (1.0%)
**Total**	**244**	**6 (2.5%)**	**1 (0.4%)**	**0.07**	**5 (2.0%)**	**0**	**1 (0.4%)**	**6 (3.2%)**

**Table 3:** Clinical and immunoserological data of anti-DFS70 positive patients.

	ID	Sex	Age	Disease	DFS IIF (titer)	DFS70 CIA (CU)	DFS70 LIA	aCL	β2GPI	LAC	Genetic anomaly
1	NI35	F	58	APS	1:80	10.8	neg	**pos**	neg	neg	neg
2	NI47	F	66	APS	1:320	0	neg	**pos**	neg	neg	neg
3	NI48	F	43	APS	1:160	0	neg	**pos**	neg	neg	neg
4	PN03	F	40	APS	neg	**23.9**	neg	**pos**	neg	—	—
5	PN23	F	38	APS	1:640	0	neg	**pos**	**pos**	—	—
6	PE20	F	42	APS	1:320	0	neg	**pos**	**pos**	—	—
7	BA15	M	37	APS	1:160	1.2	neg	**pos**	**pos**	**pos**	—
8	BA23	F	44	APS	1:640	0.2	neg	**pos**	neg	—	—
9	BA63	F	21	APS	1:320	**65.6**	**pos**	**pos**	neg	**pos**	—
10	SI13	F	76	AT	1:640	0	neg	—	—	—	—
11	SA08	M	57	AT	1:80	0	neg	—	—	—	—
12	SA20	M	92	AT	neg	**33.1**	neg	—	—	—	—
13	SA22	F	85	AT	1:80	0	neg	—	—	—	—
14	BA50	M	59	AT	1:320	0.2	neg	**pos**	**pos**	**pos**	—
15	NA8	F	46	RPL	1:640	**48.8**	**pos**	**pos**	neg	—	**MTHFR homoz**
16	NA13	F	23	RPL	1:1280	**233.9**	**pos**	**pos**	**pos**	—	**MTHFR homoz**
17	NA24	F	32	RPL	1:80	0	neg	neg	neg	—	—
18	NA34	F	38	RPL	1:160	0	**pos**	**pos**	—	—	—
19	NA35	F	43	RPL	1:80	0	neg	**pos**	—	—	—
20	PE18	F	27	RPL	1:320	0	neg	neg	neg	neg	—
21	PA25	F	30	RPL	1:640	**450**	**pos**	neg	neg	neg	neg
22	PO03	F	37	RPL	1:2560	**309.5**	**pos**	neg	neg	neg	neg
23	PO12	F	31	RPL	1:1280	**157.5**	**pos**	neg	neg	neg	neg
24	PO13	F	45	RPL	1:640	0	neg	neg	neg	neg	neg
25	SI08	M	63	Thromb. on heparin	1:160	**24**	**pos**	—	—	—	—
26	GE57	F	53	VTE	1:1280	**45.8**	**pos**	—	—	—	—
27	SI06	F	45	VTE	1:160	**24.6**	**pos**	—	—	—	neg
28	SA04	F	42	VTE	1:160	0	**pos**	—	—	—	—
29	SA49	F	45	VTE	1:320	13.4	neg	—	—	—	**MTHFR homoz**
30	TA05	F	59	VTE	1:80	0	neg	—	—	—	—
31	BA42	F	79	VTE	1:80	0.1	neg	neg	neg	**pos**	neg
32	BA74	F	63	VTE	1:80	0.2	neg	neg	neg	neg	neg
33	SB04	F	82	VTE	1:160	0	neg	neg	neg	neg	neg
34	SB36	F	80	Control on heparin	1:80	0	neg	neg	neg	neg	neg
35	SB42	F	89	Control on heparin	1:160	0	neg	—	—	—	—
36	SB56	F	75	AF	1:160	0	neg	—	—	—	—
37	SB61	F	72	AF	1:80	**43.8**	**pos**	—	—	—	—
38	SB67	F	80	AF	1:80	0	neg	—	—	—	—
39	BG41	F	58	Healthy donor	1:80	3	neg	—	—	—	—

(DFS, dense fine speckled; IIF, indirect immunofluorescence; CIA, chemoluminescence immunoassay; CU, chemiluminescence units; LIA, line-immunoassay; aCL, anticardiolipin antibodies; β2GPI, β2 glycoprotein I; LAC, lupus anticoagulant; APS, antiphospholipid syndrome; AT, arterial thrombosis; VTE, venous thromboembolism; RPL, recurrent pregnancy loss; AF, atrial fibrillation).

Women were significantly more prevalent in anti-DFS70 antibody positive patients than in anti-DFS70 antibody-negative patients according to IIF (*p* = 0.001), but not to CIA (*p* = 0.17). Anti-DFS70 positive women were younger than anti-DFS70 negative ones both for IIF (*p* = 0.04) and for CIA (*p* = 0.001).

aCL (IgG and/or IgM; cutoff 40 GPL-MPL/ml) were detected in 157 of the 294 (53.4%) patients with thrombotic events or RPL that were tested for these antibodies; anti-β2GPI (IgG and/or IgM; cutoff 10 units/ml) in 53/196 (27%), and LAC in 45/173 (26.1%). aCL, anti-β2GPI antibody and LAC frequency was not different between anti-DFS70 antibody positive and negative patients (*p* = 0.54; 0.72; and 0.44, respectively, for IIF and *p* = 0.60; 0.64; and 0.61, respectively, for CIA) (Table 4).

**Table 4:** Characteristics of anti-DFS70 positive and negative patients by chemiluminescence immunoassay CIA.

	No.	*DFS70* + *(CIA)*	*DFS70 - (CIA)*	*P*
No.	687	12	675	—
Median age (years) all cases		46.2	59.4	**0.01**
Gender F/M	456/231	10/2	446/229	0.17
Median age in F (years)		39	61	**0.001**
aCL positive (on 294 tested)	157	4/7 (57.1%)	153/287 (53.3%)	0.60
β2GPI positive (on 239 tested)	53	1/5 (20%)	52/234 (22.2%)	0.64
LAC positive (on 173 tested)	45	1/4 (25%)	44/169 (26.0%)	0.61
On heparin therapy	72	1/72 (1.4%)	71/72 (98.6%)	0.37
Genetic anomaly	32	2/32 (6.2%)	30/32 (93.8%)	0.33

Among patients treated with heparin, 1/27 (3.7%) was DFS70 positive (both in IIF and in CIA, confirmed by LIA) and 2/45 (4.4%) were positive among controls (both positive in IIF and negative in CIA and LIA) (*p* = 0.68; ns). In the group of patients that were tested for the presence of a genetic abnormality predisposing to thrombosis, 28/167 (16.8%) had a genetic mutation: nine were heterozygous for factor V Leiden, 10 had prothrombin G20210A mutation, three had homozygous MTHFR mutation with hyperhomocysteinemia, two had factor V Leiden heterozygosity and hyperhomocysteinemia, three had a deficit of protein S and one had prothrombin G20210A mutation and protein C deficiency. Two of these 28 patients (one with RPL and one with VTE) had anti-DFS70 antibodies (one detected only by IIF and one by both IIF and CIA, confirmed by LIA) without significant difference (*p* = 0.55 for IIF and *p* = 0.33 for CIA).

## Discussion

Anti-DFS70 has been defined as the enigmatic antibody^30^. Indeed, although anti-DFS70 antibodies have been thoroughly studied, their clinical association and meaning remain elusive, as their presence does not appear to be linked to any autoimmune disease, but rather to still-undefined events or causes. The recent report by Marlet^26^, which suggested the possibility that these antibodies could be associated with thrombosis or RPL, presented an intriguing hypothesis worthy of being studied more in depth. Therefore, in this study, we investigated a broad cohort of patients with venous or arterial thrombosis or with recurrent pregnancy loss, to ascertain whether an association of anti-DFS70 antibodies with thrombotic events or RPL could be confirmed by a DFS70-specific CIA method. Our data show that, when relying on IIF findings, the overall prevalence of the DFS pattern was 7%, which is consistent with the frequency found in many other diseases and approximately half the frequency reported by Marlet (this could be explained by the different selection criteria; a part of Marlet’s patients in the group of patients with thrombosis came from the hematology department to investigate unexplained thrombosis). In our series, the highest frequency as detected by IIF was observed in patients with RPL (9.9%), followed by APS (6.9%), VTE (6.1%) and arterial thrombosis (5.8%). However, when the same samples were analyzed with a DFS70-specific CIA method, only five (5.0%) of RPL, one APS (0.9%), two (1.5%) VTE and one (1.4%) arterial thrombosis were confirmed DFS70 positive.

The use of a third independent DFS70-specific LIA method indicated that all CIA-positive samples but two (those that were IIF-negative), contained antibodies targeting the DFS70 antigen.

If the ANA-IIF test alone is considered, an association between thrombotic events and the presence of anti-DFS70 antibodies is confirmed (*p* = 0.01). However, if the more specific CIA assay is considered independently from IIF results, no significant correlation is observed (*p* = 0.06). Likewise, if we consider only samples that were confirmed anti-DFS70 positive by LIA (10/12 by CIA and 2/37 by IIF), no association between thrombotic events or RPL and anti-DFS70 antibodies was apparent (*p* = 0.08).

A possible explanation for the discrepancy between IIF and CIA/LIA results, showing a higher prevalence of DFS70-like pattern by IIF, may reside in the fact that not all antibodies displaying a DFS pattern on HEp-2 cells specifically target the DFS70/LEDGFp75 antigen. Indeed, it has been shown that the DFS70/LEDGF75 protein has many cellular interacting partners which co-localize in the nucleus and that autoantibodies other than anti-DFS70 can produce a similar ANA-IIF pattern^31,32^. This pattern, recently defined as pseudo-DFS70^33^, may explain the higher positive rate of supposed anti-DFS70 antibodies detected by IIF, both in Marlet’s study, as in ours, compared to the CIA and LIA methods. Our results underscore once again that the data on the prevalence of anti-DFS70 antibodies reported in the literature are greatly influenced by the analytical method used^34^. Hence, the importance of using specific methods to avoid interpretative errors capable of misleading clinical interpretation.

Other interesting data emerge from our study. Since it has been reported that anti-DFS70 are more prevalent in younger people^34^ and especially in women^8,15^ of younger age^14,35^, we have matched the patients with healthy controls for sex and age: our findings confirm that anti-DFS70 antibodies detected by a specific method are more prevalent in younger women than in older women (*p* = 0.001) but not in females than males (*p* = 0.17).

Furthermore, Choi *et al*.^36^, in their study on an international inception cohort of 1137 lupus patients, found that anti-β2GPI antibodies were more frequently associated with anti-DFS70 antibodies (odds ratio 2.17) than aCL and LAC. Though our patient cohorts differ from that of Choi, we cannot confirm an association between anti-β2GPI and anti-DFS70 antibodies as only 5/53 (9.4%) patients with anti-β2GPI were anti-DFS70 positive by IIF and 1/53 (1.9%) by CIA.

Finally, since heparin has been shown to be a stabilizing factor of DFS70/LEDGF, protecting it from proteolytic degradation under various stress conditions^29^, to ascertain a possible role played by heparin treatment in anti-DFS70 expression, we included in this series of patients 25 subjects who had a venous or arterial thrombosis currently under heparin therapy and 45 subjects without thrombosis on prophylactic treatment with heparin before orthopedic surgery. Results show that heparin has no effect on DFS70 antibody production, since only 1/72 subjects on heparin treatment was found to be anti-DFS70 antibody positive by CIA.

Limitations of this study may reside on the fact that data of APS-related antibodies as well as search for genetic anomalies were not available for all patients; also the number of patients treated with heparin was small. However, to date this is the largest study on the prevalence of DFS70 antibodies in patients with APS and the first one to investigate the possible link between anti-DFS70 antibodies and heparin.

In conclusion, this study is the largest published to date on the prevalence of anti-DFS70 antibodies in patients with thrombotic events or recurrent pregnancy loss. Results obtained by specific CIA and LIA methods do not indicate that venous or arterial thrombosis or RPL are linked to a higher prevalence of anti-DFS70 antibodies. Further studies are needed to solve the DFS70 enigma.

## Data Availability

The datasets generated during and/or analysed during the current study are available from the corresponding author on reasonable request.
